# Treatment of Malrotation and Limb Length Discrepancy in Osteogenesis Imperfecta Patients: Report of Two Cases

**DOI:** 10.5704/MOJ.2203.016

**Published:** 2022-03

**Authors:** MA Ibrahim, NAF Nik-Mohamed, I Munajat, AR Sulaiman, EF Mohd

**Affiliations:** 1 Department of Orthopaedic, Hospital Sultanah Nur Zahirah, Kuala Terengganu, Malaysia; 2 School of Medical Sciences, Hospital Raja Perempuan Zainab II, Kota Bharu, Malaysia; 3 Department of Orthopaedics, Universiti Sains Malaysia, Kubang Kerian, Malaysia

**Keywords:** iliac bone graft, osteogenesis imperfecta, shortening, acute lengthening, malrotation

## Abstract

Malunion of recurrent fractures in Osteogenesis Imperfecta (OI) patients causes limb length discrepancy and malrotation. These cause added difficulty for OI patients to ambulate. Lengthening with distraction osteogenesis using an external fixator in OI patients is challenging. Acute lengthening with autologous bone graft is a known method in a normal bone but not a known procedure in OI patients. We present two clinic cases of adolescent OI patients with limb length discrepancy and externally rotated lower limb that underwent acute lengthening and rotational correction using a locked intramedullary nail and ipsilateral autologous iliac bone graft. Both patients obtained union and improvement of ambulatory capability without recurrence of fracture within five years of follow-up. Acute lengthening by 2cm and rotational correction with intramedullary nail improved the gait efficiency in the OI patients. Harvesting large amounts of the tricortical iliac bone graft, followed by controlled weight-bearing is a safe procedure.

## Introduction

An osteogenesis imperfecta (OI) patient has ambulatory difficulty due to bone deformity, recurrent fractures, weak muscles, and fear of ambulating^1^. Limb length discrepancy (LLD) and malrotation pose additional challenges. The LLD can be attributed to angular deformity, growth arrest, malunion, and multiple previous fractures^2^.

Malrotation due to malunion following improper external splinting or the inability of an intramedullary device like the rush rod to prevent rotational deformation during a healing process is rarely reported in the literature for OI patients. External malrotation of femur leads to knee malorientation and a posterior shift in the weight bearing axis in the sagittal plane^3^. Correction of the deformity with acute distraction and autologous bone graft is common in a typical patient but not well reported in the English literature for OI patients.

We report two successful mature OI cases of acute lengthening and rotational correction of femoral deformity with autografts and interlocking nail.

## Case Report

**Fig. 1:**
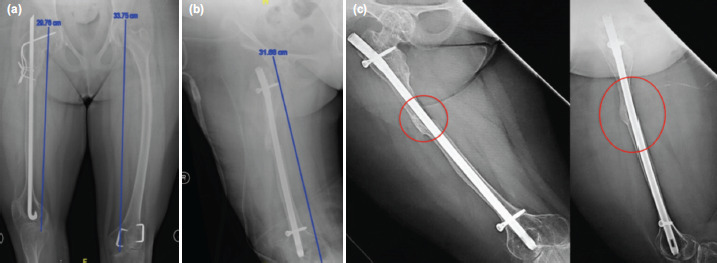
(a) Pre-operative radiograph with estimated 4cm shortening and malrotation, evidence by the overlapping distal femoral condyles in anteroposterior view. (b) Post-operative radiographs with bone gap of 2cm filled with iliac bone cortical graft. (c) Post-operative AP and Lateral radiograph showing well incorporated bone graft.

The first patient was a 16-year-old OI Sillence type IV female patient who presented with an inability to ambulate properly since the age of ten following a fracture of the right femur. Examination revealed 4cm right femur shortening. The hip external rotation ranged from 10° - 110° with a 70° externally rotated femur. A radiograph showed a rush rod and K-wires in situ (Fig. 1a).

**Fig. 2:**
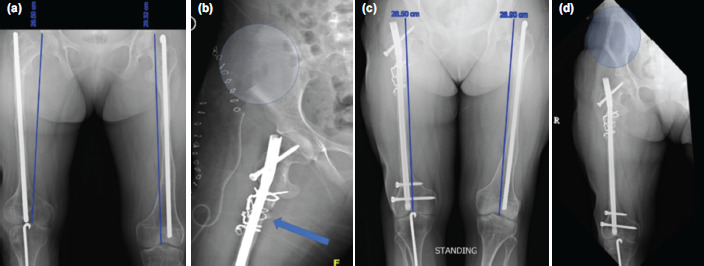
(a) Pre-operative radiograph with estimated 3cm shortening and malrotation evidence by obliquely overlapped distal femoral condyle, patella facing laterally, and the fibula lies behind the tibia. (b) Post-operative radiograph showing bone graft at the subtrochanteric region stabilised with cerclage wire (arrow) with large area of tricortical graft defect (circle). (c) Post-operative radiograph with well incorporation of the bone graft with acceptable length. (d) Healing of the iliac donor site (circle) and well incorporated femur graft.

The second patient was an 18-year-old OI Sillence type IV female patient with previous multiple fractures and surgeries fixed with Rush rod. She presented with an out-toeing gait and difficulty walking. Examination revealed 3cm shortening and 60° externally rotated right femur. Radiographs showed coxa vara of the right femur and externally rotation with rush rod in situ (Fig. 2a). Both patients have muscle wasting but normal power and neurology of the lower limb.

Surgical procedures started with implant removal and femoral osteotomy (at midshaft in case 1 and subtrochanteric in case 2). Then we performed retrograde proximal reaming with a cannulated drill bit, aimed towards the piriformis fossa, followed by control rigid hand reaming with gradual increment until size 1m larger than the proposed nail size. We repeated the similar antegrade reaming till distal. We insert the selected size of the intramedullary nail, passing the osteotomy site until the superior pole of the patella. We rotated distal fragment 60° internally and inserted the distal locking screws. Following that, the femur was distracted for 2cm, and then the proximal screws inserted. Finally, we harvested an ipsilateral tricortical iliac bone graft, inserted it into the distracted area, and secured it with either a nonabsorbable suture (Fig. 1b) or cerclage wire (Fig. 2b)

Post-operatively, there was no neurological deficit. Both patients were put on non-weight bearing for six weeks, followed by partial weight-bearing with self-strengthening and motion exercises. Only case 2 received bisphosphonates treatment as continuation of the previous regime. Eventually, the distracted osteotomy site united, the bone graft was incorporated (Fig. 1c and Fig. 2c), the donor site healed without complication, and the patient walked unaided with an acceptable foot progression angle (Fig. 2d). Both patients obtained union and improvement of ambulatory capability without recurrent fracture within five years of follow-up.

## Discussion

Lengthening by distraction osteogenesis with external fixation in an already abnormal bone is associated with complications, and it is more appropriate to use an intramedullary nail^2^. We did not choose the prior technique as it carries the risk of infection and requires prolonged rehabilitation.

Acute lengthening together with rotational correction over interlocking intramedullary devices is an option. This method was chosen for these patients as it provided immediate stability and allowed early rehabilitation that prevents disuse osteoporosis in already weak OI bone. Current intramedullary devices for children lack a locking screw for rotational control. The delay in surgical correction is for the bone length and diameter growth to accommodate the size of the currently available implant. The ideal implant would be a custom-made locking nail with adequate fixation to the neck and shaft of the femur to prevent recurrent fracture and control rotation.

Acute lengthening over the intramedullary nail up to 3.5cm or 12.6% was safe in a typical patient^4^. Excessive acute lengthening might lead to neurovascular injury, delayed union, or non-union. Since our patients had short right femur, we performed 2cm acute lengthening, equal to 6.8% of its original length, to avoid complications. The final length gained in case no. 2 was more than 3cm after 2cm of acute distraction due to the contribution of the coxa vara correction (Fig. 2c). In both patients, the acute distraction performed was safe without causing any complication to the nerves, vessels, muscles, or joints. Life-long protection was planned for this patient to avoid recurrent fracture and possible nonunion.

Puvanesarajah *et al* reported the successful use of allograft in treating non-union in OI patients^5^. To our knowledge, there has been no report in English literature of using iliac bone graft in OI patients. The ipsilateral iliac bone graft harvested site was planned away from the body load to optimise weight bearing on the contralateral side. A fracture is possible if the ipsilateral side iliac bone graft was taken, and the patient might not be allowed partial weight-bearing ambulation. The waiting period of the controlled loading was until there was radiological evidence of a new bone formation (Fig. 2d).

Even though malrotation of the long bones in OI patients following recurrent fractures is not well reported, malrotation is a known complication following a femoral fracture treated non-operatively or with nonlocking intramedullary nail. Malrotation of more than 30° disturbs walking function, and external malrotation is less tolerable^3^. Both of our patients had more than 60° external malrotation. Acute correction up to 60° on top of acute lengthening of 2cm did not cause neurovascular compromise in these two patients.

In conclusion, acute lengthening by 2cm and rotational correction with intramedullary nail improved the gait efficiency in the OI patients. Harvesting large amount of the tricortical iliac bone graft, followed by a controlled weight-bearing is a safe procedure. The iliac donor side regrew without evidence of fracture, with controlled weight-bearing, and away from the body load.
